# Roles of Glycoproteins in the Diagnosis and Differential Diagnosis of Chronic and Latent Keshan Disease

**DOI:** 10.3390/molecules22050746

**Published:** 2017-05-08

**Authors:** Sen Wang, Zheng Fan, Bing Zhou, Yingting Wang, Peiru Du, Wuhong Tan, Mikko J. Lammi, Xiong Guo

**Affiliations:** 1 School of Public Health, Health Science Center of Xi’an Jiaotong University, Xi’an 710061, Shaanxi, China; senwang2016@163.com (S.W.); wytlyr0411@stu.xjtu.edu.cn (Y.W.); dpr1212@stu.xjtu.edu.cn (P.D.); guox@xjtu.edu.cn (X.G.); 2 Office of Teaching Affairs, Xi’an University, Xi’an 710061, Shaanxi, China; xiaofandd@163.com; 3 Key Laboratory of Hormones and Development (Ministry of Health), Key laboratory of metabolic disease (Tianjin), Metabolic Diseases Hospital & Tianjin Institute of Endocrinology, Tianjin Medical University, Tianjin 300070, China; duduyu2016@126.com; 4 Department of Integrative Medical Biology, University of Umeå, Umeå 90187, Sweden

**Keywords:** lectin microarray, Keshan disease, biomarker, glycoprotein, saliva, serum

## Abstract

We aimed to explore the roles of glycoproteins in the pathogenesis of chronic and latent Keshan disease (CKD and LKD), and screen the lectins as indicators of significant differences in glycoproteins of KD saliva and serum. Blood and saliva were collected from 50 CKD, 50 LKD patients and 54 normal individuals. Saliva and serum lectin microarrays and saliva and serum microarrays were used to screen and verify the differences in the levels of lectin among the three groups. In the male saliva lectin microarray, *Solanum tuberosum* (potato) lectin (STL) and other 9 lectins showed differences between CKD and normal; STL and other 9 lectins showed differences between LKD and normal; *Aleuria aurantia lectin* (AAL) and other 15 lectins showed differences between CKD and LKD. In the female saliva microarray, *Griffonia (Bandeiraea) simplicifolia lectin I* (GSL-I) and other 9 lectins showed differences between CKD and normal; STL and other 7 lectins showed differences between LKD and normal; *Maackia amurensis lectin I* (MAL-I) and *Triticum vulgaris* (WGA) showed difference between CKD and LKD. In the male serum lectin microarray, *Psophocarpus tetragonolobus lectin I* (PTL-I) and other 16 lectins showed differences between CKD and normal; Ulexeuropaeus agglutinin I (UEA-I) and other 9 lectins showed differences between LKD and normal; AAL and other 13 lectins showed differences between CKD and LKD. In the female serum lectin microarray, WGA and other 13 lectins showed differences between CKD and normal; *Euonymus europaeus lectin* (EEL) and other 6 lectins showed differences between LKD and normal; MAL-I and other 14 lectins showed differences between CKD and LKD. Carbohydrate chain GlcNAc and α-Gal may play crucial roles in the pathogenesis of KD. STL may be considered the diagnostic biomarker for male CKD and LKD, while WGA may be useful in distinguishing between the two stages. STL may be considered the diagnostic biomarker for female LKD.

## 1. Introduction

Keshan disease (KD) is an endemic, highly lethal cardiomyopathy, which was first reported in northeast China’s Keshan county in 1935. Later, there were similar reported cases of KD in Japan’s Nagano prefecture and in the northern mountains of North Korea in the 1950s [1]. The clinical features are acute or chronic episodes of heart disorder characterized by cardiogenic shock, arrhythmia, and congestive heart failure, with cardiomegaly. KD affected 40,088 residents in 16 provinces, according to the 2010 Statistical Yearbook of Health in China [2]. Keshan disease patients are categorized clinically into four groups: acute, sub-acute, chronic, and latent. The disease is characterized by multifocal myocardial necrosis and fibrosis that leads to cardiogenic shock and congestive heart failure. Chronic KD (CKD) and latent KD (LKD) are the most two common types [3]. CKD: the onset is slow. The patients present chronic heart failure and dilated chambers of the hearts; the heart walls become thinner than normal, and there is a widespread myocardial fibrosis. LKD: the onset is disguised, and the patients have reasonably good heart function (NYHA class I). Ventricular extrasystole and right bundle branch block or ST–T change are common. Cardiomegaly is not observed [4].

Protein glycosylation plays a key role in various biological processes, such as maintaining normal cell functions, development, intercellular signaling, protein–protein interactions, protein folding, metabolism, bacterial infections, cellular differentiation, tumor metastasis and the pathogenesis and progression of various diseases, among others. Approximately half of human proteins are thought to be modified through diverse glycosylation patterns [5]. Lectins are carbohydrate-binding proteins that have been used to discriminate glycans based on subtle differences in structure, including glycoproteins, glycolipids and glycosaminoglycans, in a rapid, precise and high-throughput manner [6]. Oligosaccharides present in the glycoproteins of human whole saliva and serum include, for example, galactose, amino sugars, mannose and sialic acids (Sia). Proteins that perform many different functions are modified by the carbohydrates [7]. The Sialic acids (Sia) are a group of saccharides that provide many functions to glycoproteins in a wide range of organisms, mainly in higher eukaryotes [8].

Lectin microarrays enables simultaneous observations of multiple distinct binding interactions and has become one of the main ways to investigate glycosylation patterns [9]. The lectin microarray method was established and validated for the rapid analysis of glycoproteins in a previous paper [6]. This method can be used in the study of the relationships between the glycoproteins and cancer, diabetes mellitus, hepaticcirrhosis and some age related diseases [10].

The etiology of KD remains unclear. The pathogenesis has not yet been fully defined. Currently, indicators for differential diagnosis based on the glycosylation levels are not available for CKD and LKD. Saliva and serum which are collected easily with no or less trauma are suitable to be applied to diagnosis. In this study, we focused on revealing the changes in the glycosylation characteristics of the saliva and serum among the CKD and LKD patients. The lectin microarrays were used to screen the expression level of various glycoprotein carbohydrate structures. Protein binding microarray was used to verify the result of lectin microarray. Finally, this study aimed to screen potential lectins as differential diagnostic biomarkers of CKD and LKD patient using saliva and serum. The prevalence rate of KD in females is higher than in males [2], so we analyzed the glycoprotein and lectin-binding profiles in the female and the male patients separately.

## 2. Results

### 2.1. Male Saliva Lectin Microarray

Specificity carbohydrate chains recognized by 37 lectins and their full names were shown in Table 1.

**Figure 1:**
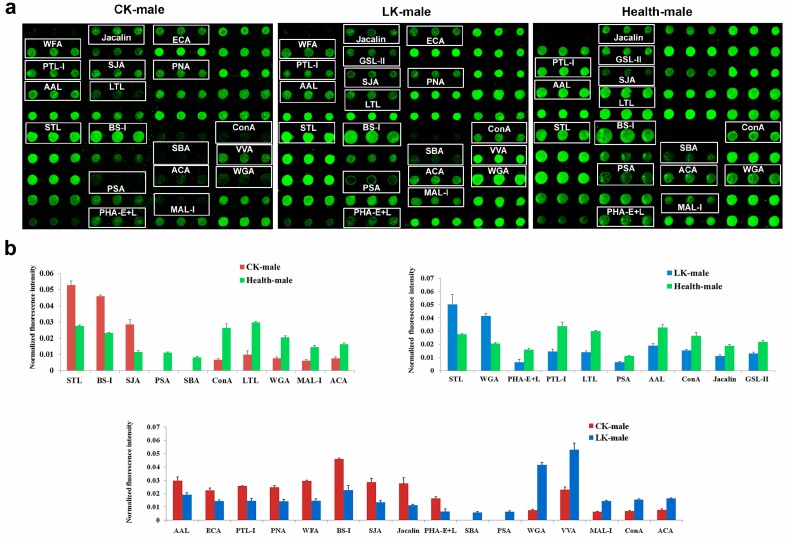
Male salivary glycoprotein glycosylation patterns were determined by the binding of Cy3-labeled protein to 37 different lectins using a lectin microarray. CK: CKD, LK: LKD and Health: normal control. (**a**) Profiles of Cy3-labeled salivary proteins from patients with CKD, LKD, and normal control individuals bound to the lectin microarrays. The lectins, which exhibited remarkable differences in the staining intensity are marked with white frames; (**b**) Comparisons of the lectin-binding levels between the three groups. The bars represent mean ± SD measured from three replicate spots of lectins.

The lectin microarray analysis of the saliva showed significant differences in the fluorescence intensities of 10 lectins between the male CKD and male normal control groups. In the CKD group, the lectins STL, BS-1 and SJA bound higher amount of Cy3-labeled proteins than normal controls. However, the signals of PSA, SBA, ConA, LTL, WGA, MAL-I and ACA decreased in the male patients with CKD compared to the normal group (Figure 1).

In the male LKD group, significant differences were observed in the fluorescence intensities of 10 lectins in comparison to the male normal control group. Stronger than control signals were measured for STL and WGA, while PHA-E+L, PTL-I, LTL, PSA, AAL, ConA, Jacalin and GSL-II signals were lower in comparison to the saliva samples from the male normal control group (Figure 1).

Comparison of the CKD and the LKD profiles may reveal differences between the two stages of the KD, therefore, it was done to the CKD and the LKD groups. Significant differences in the fluorescence intensities of 16 lectins were observed between the male CKD and male LKD groups in the saliva lectin microarray. The CKD samples produced stronger signals for AAL, ECA, PTL-I, PNA, WFA, BS-I, SJA, Jacalin and PHA-E+L than the LKD ones, while SBA, PSA, WGA, VVA, MAL-I, ConA and ACA signals were (Figure 1).

### 2.2. Lectin Microarray Analysis of the Female Saliva

**Figure 2:**
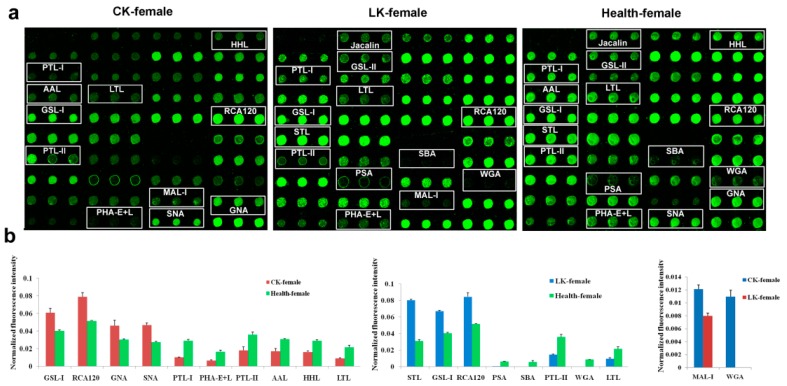
Female salivary glycoprotein glycosylation patterns were determined by the binding of Cy3-labeled protein to 37 different lectins using a lectin microarray. CK: CKD, LK: LKD and Health: normal control. (**a**) Profiles of Cy3-labeled salivary proteins from patients with CKD and LKD, and normal individuals bound to the lectin microarrays. The lectin microarrays revealed lectins that exhibited significant differences, which are marked with white frames; (**b**) Significant differences in the lectin levels among the three groups. The bars represent mean ± SD measured from three replicate spots of lectins.

Lectin-binding analysis of female salivary proteins yielded a different set of differential expression of the lectins, although some similarities were present, too. In the CKD, *s*ignificant differences in the fluorescence intensities of 10 lectins were noticed between the female CKD and female normal groups. The stronger signals for CKD samples were in GSL-I, RCA120, GNA and SNA. Meanwhile, the PTL-I, PHA-E+L, PTL-II, AAL, LTL and HHL signals decreased in the CKD saliva compared to the normal group (Figure 2).

In the LKD, significant differences in the fluorescence intensities of 8 lectins were observed between the female LKD and female normal groups in the saliva lectin microarray. Higher signals were present in STL, GSL-I and RCA120 in LKD compared with the normal control group. Meanwhile, the PSA, SBA, PTL-II, WGA and LTL signals decreased in the patients with LKD (Figure 2).

In a comparison between the CKD and LKD, a significant difference in the fluorescence intensity was observed in MAL-I and WGA (Figure 2).

### 2.3. Lectin Microarray of the Male Serum

**Figure 3:**
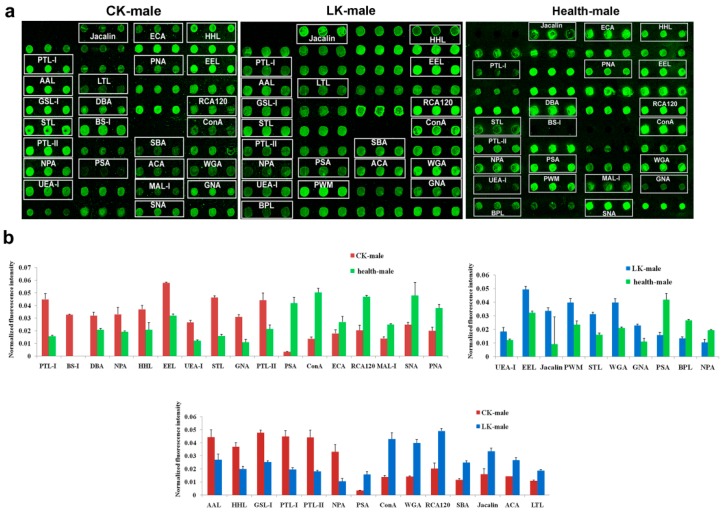
Male serum glycoprotein glycosylation patterns were determined by the binding of Cy3-labeled protein to 37 different lectins using a lectin microarray. CK: CKD, LK: LKD and Health: normal control. (**a**) Profiles of Cy3-labeled serous proteins from patients with CKD and LKD, and normal individuals bound to the lectin microarrays. The lectin microarrays revealed lectins that exhibited significant differences, which are marked with white frames; (**b**) Significant differences in the lectin levels among the three groups. The bars represent mean + SD of the three biological replicates from each group.

In the males, the serum samples displayed a wider panel of changes in lectin binding than the saliva sample. In CKD, changed fluorescence intensities in a total of 17 lectins were observed in comparison to normal control group. Increased signals were in PTL-I, BS-I, DBA, NPA, HHL, EEL, UEA-I, STL, GNA and PTL-II were observed in the serum from male patients with CKD than in the male normal group. Decreased signals were observed in PSA, ConA, ECA, RCA120, MAL-I, SNA and PNA signals (Figure 3).

In the LKD, fewer differences in male serum could be seen in comparison to the normal control group. A total of 10 lectins were observed between the male LKD, including UEA-I, EEL, Jacalin, PWM, STL, WGA and GNA as lectins, which had higher binding to the Cy3-labelled glycoproteins. Meanwhile, PSA, BPL and NPA signals decreased in the analysis of the LKD serum (Figure 3).

There were several lectins which might be useful in differentiating the CKD and the LKD in the male. Significant differences in the fluorescence intensities of 14 lectins were revealed between the CKD and LKD groups. Stronger signals in the CKD were acquired for AAL, HHL, GSL-I, PTL-I, PTL-II and NPA, while PSA, ConA, WGA, RCA120, SBA, Jacalin, ACA and LTL produced weaker signals in the CKD than in the LKD group (Figure 3).

### 2.4. Lectin Microarray Analysis of the Female Serum

**Figure 4:**
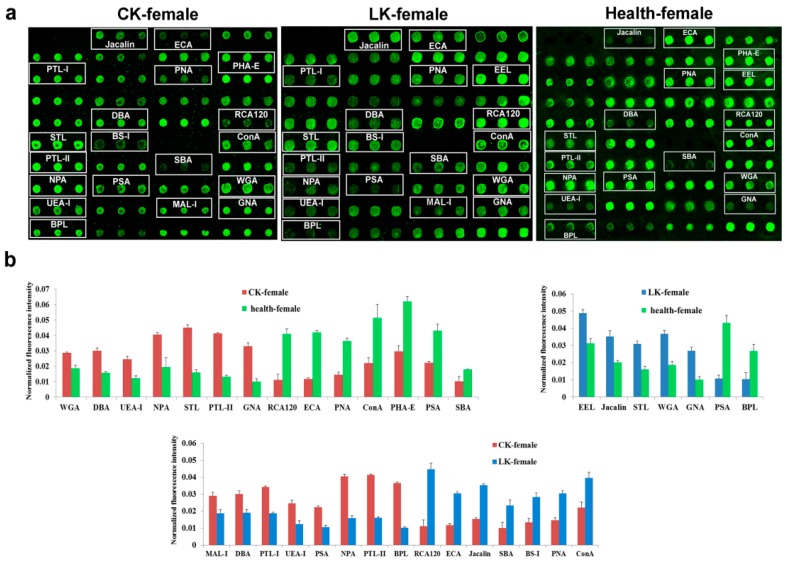
Female serum glycoprotein glycosylation patterns were determined by the binding of Cy3-labeled protein to 37 different lectins using a lectin microarray. CK: CKD, LK: LKD and Health: normal control. (**a**) Profiles of Cy3-labeled serous proteins from patients with CKD, and LKD, and normal individuals bound to the lectin microarrays. The lectin microarrays revealed lectins that exhibited significant differences, which are marked with white frames; (**b**) Significant differences in the lectin levels among the three groups. The bars represent mean + SD of the three biological replicates from each group.

In the female CKD serum, we could see significant differences in 14 lectins in relation to the normal control group. The increased fluorescence intensities were measured for WGA, DBA, UEA-I, NPA, STL, PTL-II and GNA. On the other hand, those for RCA120, ECA, PNA, ConA, PHA-E, PSA and SBA were decreased (Figure 4).

In the LKD, patterns for 7 lectins were different. Binding of the Cy3-labelled glycoproteins to EEL, Jacalin, STL, WGA and GNA were increased compared to the normal control group, simultaneously to decreased binding to PSA and BPL (Figure 4).

Altogether 15 lectins were differentially expressed between the CKD and the LKD groups. Stronger signals for MAL-I, DBA, PTL-I, UEA-I, PSA, NPA, PTL-II and BPL were observed in the serum from the female CKD patients than that from the patients with LKD. In contrast, RCA120, ECA, Jacalin, SBA, BS-I, PNA and ConA signals weaker the CKD serum (Figure 4).

### 2.5. Saliva Microarray

**Figure 5:**
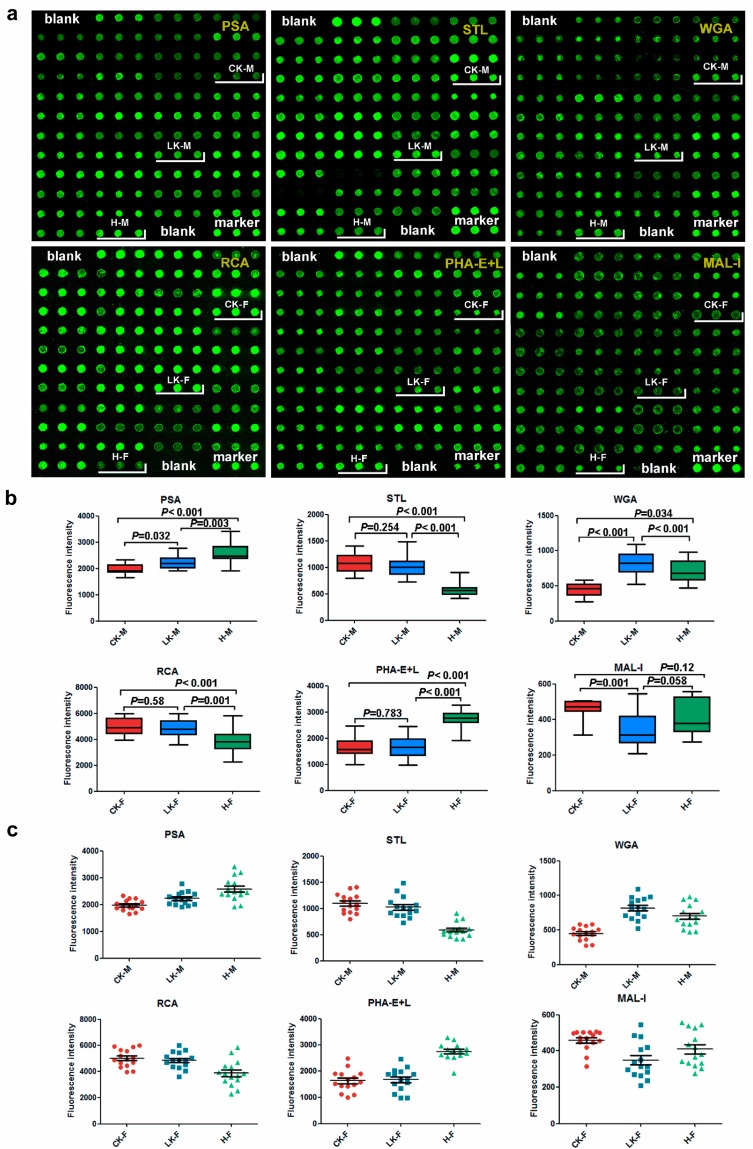
Validation of the binding of 6 lectins (PSA, STL, WGA, RCA, PHA-E+L and MAL-I) to saliva glycoproteins. (**a**) Scanned images of Cy3-labeled lectins bound to the saliva microarrays; (**b**) Box plot analysis of the data for the six groups obtained from the salivary microarray. Error bars represent 95% confidence intervals for mean values. The statistical significance of differences between groups is indicated by the *p*-value. (**c**) Scatter plot analysis of the data for the six groups obtained from the saliva microarray. Lines represent mean ± SEM. CK-M: male patients with CKD, LK-M: male patients with LKD, H-M: male normal controls, CK-F: female patients with CKD, LK-F: female patients with LKD and H-F.

Based on lectin microarray results, 6 lectins were chosen for saliva protein microarray to validate the results with 15 individual samples per group. Data from the male saliva microarray showed a statistically significant decrease in PSA signal of the CKD group compared to the male normal control one. A significant decrease was also noticed in the LKD group, although it also differed from the CKD group (Figure 5). A stronger STL signal was observed in the saliva of the male CKD and LKD than in those of the male normal controls. However, no significant difference in the STL signal was not observed between the saliva samples from the male CKD and the LKD groups (Figure 5). The WGA signal decreased in the saliva from the male CKD group compared to the male normal group. Meanwhile, the WGA signal also decreased in the saliva from the male normal group compared to the male LKD group (Figure 5).

In the female saliva microarray, a stronger RCA signal was observed in the saliva from the female CKD and LKD patients than in those from the female normal controls. However, the CKD and LKD groups did not significantly differ from each other. The PHA-E+L signal was significantly lower in the saliva from the female CKD and LKD groups compared to the normal control group. However, significant difference in the PHA-E+L signal was not observed between the CKD and the LKD groups. Signal for MAL-I was significantly lower in the saliva from the LKD patients in comparison to the CKD group (Figure 5).

### 2.6. Serum Microarray

**Figure 6:**
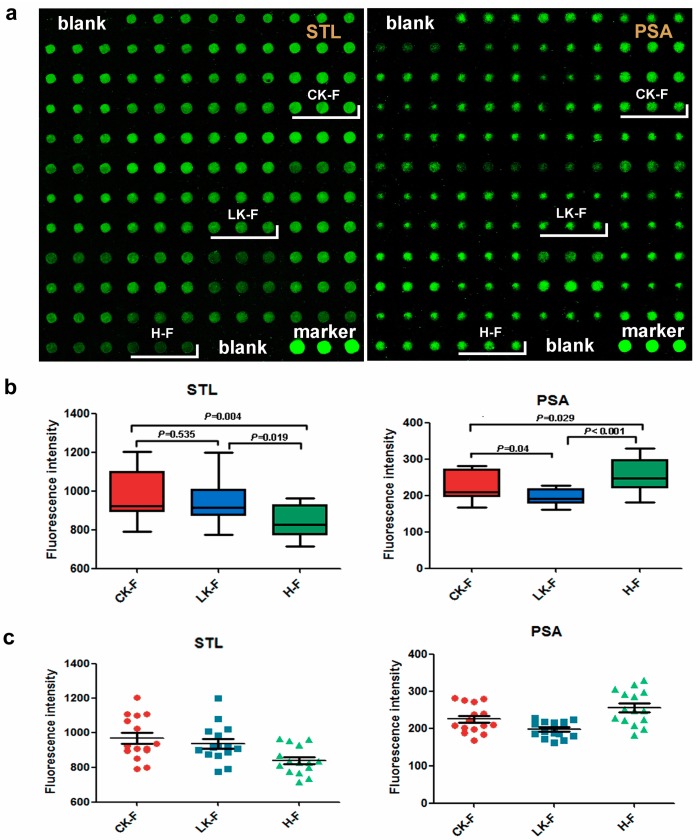
Validation of the binding of 2 lectins (STL and PSA) to serum glycoproteins. (**a**) Scanned images of Cy3-labeled lectins bound to the serum microarrays; (**b**) Box plot analysis of the data for the three groups obtained from the serum microarray. Error bars represent 95% confidence intervals for mean values. The statistical significance of differences between groups is indicated by the *p*-value. (**c**) Scatter plot analysis of the data for the six groups obtained from the saliva microarray. Lines represent mean ± SEM. CK-M: male patients with CKD, LK-M: male patients with LKD, H-M: male normal controls, CK-F: female patients with CKD, LK-F: female patients with LKD and H-F.

In the female serum microarray, STL signal was increased in the serum samples from the CKD and LKD patients in comparison to normal controls. The PSA signal decreased significantly in the serum samples from the LKD group compared to both normal control and the CKD groups (Figure 6).

## 3. Discussion

Glycosylation is one of the most common posttranslational modifications of secreted proteins and plays a significant role in cell-cell interactions, cell adhesion, malignant transformation and metastasis. The glycopatterns of glycoproteins provide clues about cell metabolism, expression and function of oligosaccharides. Saliva is a good indicator of the levels of various substances in serum [11]. Both the saliva and the serum samples were utilized in this study to test whether variation in the glycosylation patterns of their glycoproteins would be useful to indicate the CKD and the LKD, and perhaps even provide a tool to distinguish them.

Based on both the saliva and the serum, a stronger STL signal was observed from the male patients with CKD compared with the normal group; stronger STL and WGA signals were observed in the the male LKD compared with the normal group; a weaker WGA signal was observed in the male CKD compared with the LKD group; and a stronger STL signal was observed in the female LKD compared with the normal group.

(*O*-GlcNAc) covalent modification of cardiac signaling proteins has gained significant attention as a key player in cardiac pathology [12]. A role for *O*-GlcNAcylation in the regulation of cardiomyocyte excitation–contraction coupling has been demonstrated [13]. Intracellular Ca^2+^ homeostasis is also directly influenced by *O*-GlcNAcylation; elevated levels of *O*-GlcNAc attenuate-induced increase in cardiomyocyte diastolic Ca^2+^ levels [14]. Furthermore, there is an indication that cardiac autophagy and apoptosis proteins, Beclin-1 and Bcl-2, are also modified by *O*-GlcNAc suggestive of a role in cell death signaling [15]. Recent evidence suggests that *O*-GlcNAcylation of key regulators of hypertrophic gene expression, HDACs, mSin3A and repressor element-1 silencing transcription factor, is important in exercise-induced cardiac hypertrophy [16]. Post-translational protein modification by GlcNAc on serine or threonine residues called *O*-GlcNAcylation is an important mechanism to protect the myocardium [17]. Increased *O*-GlcNAc protein levels increase cell survival following stress. Numerous studies have shown that acute induction of the HBP and *O*-GlcNAc protein modification protects cells from a variety of stresses, including heart disease [18]. GlcNAc particles reduced aberrant calcium release in failing cardiac myocytes and restored sarcomere function [19]. *O*-GlcNAcylation was increased in an infarct-induced heart failure model. Several in vitro and in vivo analyses showed *O*-GlcNAc-mediated cardioprotection against ischemia-reperfusion, myocardial infarction, and oxidative stress [20]. GlcNAc may be an important player in the regulation of many key functional processes in cardiomyocytes and may play a crucial epigenetic role in mediating pathological outcomes. It has been demonstrated that α-Gal antigen was associated with myocardial fibroblasts, cardiomyocytes and blood vessels in the native porcine myocardium [21].

Carbohydrate chain GlcNAc and α-Gal which are associated with cardiomyocyte may play key roles in pathogenesis of KD. Lectins GSL-II, PHA-E, STL, WGA, PWM and BS-I, which are the indicators of the significant glycoproteins mentioned above, are associated with cardiomyocyte.

This study indicates that lectin-based analysis can be a valuable tool in the diagnosis of the CKD and the LKD. Based on both the saliva and the serum analyses, STL may be considered as the diagnostic biomarker for male CKD and LKD, while WGA may be useful in distinguishing between the two stages. STL may be considered as the diagnostic biomarker for female LKD.

## 4. Materials and Methods

### 4.1. The Study Groups

Patients with CKD (*n* = 50, 25 males, 25 females, 54–66 years old), patients with LKD (*n* = 50, 25 males, 25 females, 53–68 years old) and normal control individuals (*n* = 54, 27 males, 27 females, 55–67 years old) were recruited from Huangling County in Shaanxi Province, China.

### 4.2. Peripheral Blood and Whole Saliva Collection

Both venous blood and saliva were collected from patients with CKD, patients with LKD and the matched normal group. Three milliliters of venous blood and 3 mL of unstimulated saliva were collected at least 2 h after the last intake of food. Each venous blood sample was incubated at room temperature for 30 min. The serum was collected by centrifugation at 3000 rpm for 5 min and then used immediately or stored at −80 °C. Each participant’s mouth was rinsed with 0.9% saline before saliva was collected. A protease inhibitor cocktail (1 μL/mL of whole saliva, Sigma, St. Louis, MO, USA) was added to the saliva immediately upon collection. Whole saliva was then centrifuged at 12,000 rpm for 30 min at 4 °C to remove the insoluble material. The supernatant was collected and filtered (0.20 μm pore size) to remove bacteria and microbes and then used immediately or stored at −80 °C.

All normal controls were excluded from the patients with cardiovascular disease, diabetes and hypertension. Informed consent was obtained from each subject involved in the investigation. This investigation was approved by the Human Ethics Committee of Xi’an Jiaotong University, and performed according to the principles of the Declaration of Helsinki as revised in 1983.

### 4.3. Saliva and Serum Lectin Microarrays

Ten male and 10 female samples of saliva and serum from CKD and LKD patients, and 12 male and female samples of saliva and serum from normal controls were selected. The other 15 male and 15 female samples from CKD, LKD and normal groups were used in the saliva and serum microarrays to further validate the results.

**Figure 7:**
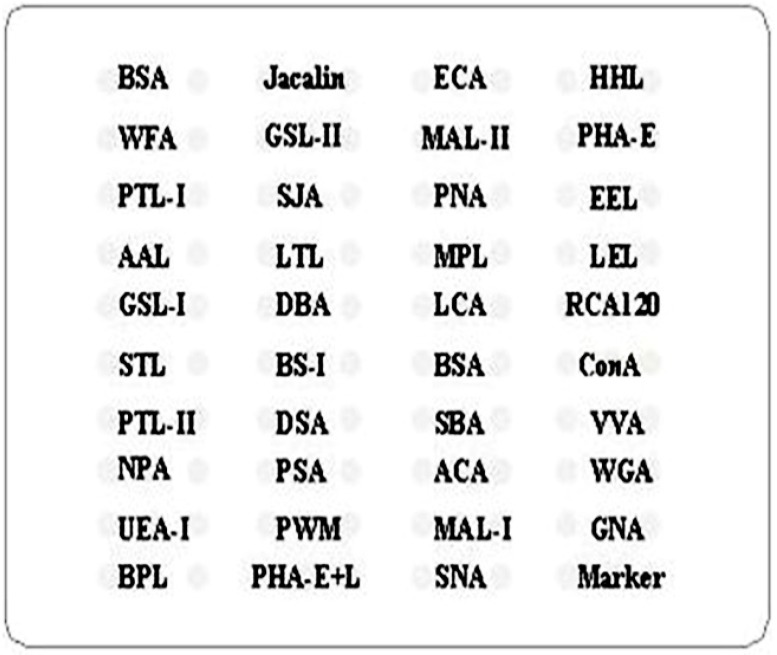
Layout of saliva or serum lectin microarray containing 37 lectins, the negative control, bovine serum albumin (BSA), and a marker.

Saliva and serum lectin microarrays were produced separately by incorporating a negative control (bovine serum albumin, BSA), a marker and 37 lectins (Sigma, St. Louis, MO, USA) with different binding preferences for N- and O-linked glycans. The layout of the lectin microarray is shown in Figure 7. The lectins weredissolved to a concentration of 1 mg/mL in buffercontaining a 1 mmol/L concentration of the appropriate monosaccharide and spotted onto the epoxysilane-coated slides with Stealth micro spotting pins (SMP-10B; TeleChem, Atlanta, GA, USA) and a Capital smart microarrayer (CapitalBio, Beijing, China) usinga previously reported protocol. Each lectin was spotted in triplicate per block. Four milligrams of Cy3-labeled salivary protein was diluted in 0.5 ml of incubation buffer containing 2% (*w*/*v*) BSA, 500 mmol/L glycine and 0.1% Tween-20 and applied to the blocked lectin microarrays. The microarrays were scanned with a 70% photomultiplier tube setting and 100% laser power using a Genepix 4000B confocal scanner (Axon Instruments Inc., Union City, CA, USA). The images were acquired at 532 nm to detect Cy3 detection and analyzed using Genepix 3.0 software (Axon Instruments Inc., Union City, CA, USA). Three replicate slides of each sample were consistently analyzed. The median of the effective data points for each lectin was globally normalized to the sum of medians of all effective data points for each lectin in one block [10].

### 4.4. Analysis of Saliva and Serum Lectin Microarray

First, we obtained the mean of fluorescence intensity value from each group (male CKD, male LKD, male normal group, female CKD, female LKD and female normal group). Then, we separately compared the two means of two groups (male CKD/male normal, male LKD/male normal, male CKD/male LKD group, female CKD/female normal, female LKD/female normal and female CKD/female LKD group). Over 1.5-fold or less than 0.67-fold changes in the fluorescence between the pairs were considered to indicate up-regulation or down-regulation of the lectin.

### 4.5. Saliva and Serum Microarrays

For the saliva and serum microarrays,15 male and 15 female patients with CKD, 15 male and 15 female patients with LKD, and 15 male and 15 female normal control individuals were used to verify the results of the saliva and serum lectin microarrays. The lectins PSA, STL, WGA, RCA, PHA-E+L and MAL-I were selected to verify the results of saliva lectin microarrays, and STL and PSA to verify the results of serum lectin microarrays. Previously reported procedures were used to analyze the saliva and serum microarrays [10]. Each sample was spotted in triplicate on the microarray. Then, the Cy3-labeled lectins were applied to detect the specific sugar structures in minimal amounts of saliva samples that were immobilized on the slides. The slide was scanned using a Genepix4000B confocal scanner(Leica, Munich, Germany), and the acquired images were analyzed at 532 nm to detect Cy3, and the acquired images were analyzed using Genepix 3.0 software (Axon Instruments Inc., Union City, CA, USA).

### 4.6. Statistical Analysis of Saliva and Serum Microarrays

Significant differences between groups were calculated using one-way ANOVA with SPSS statistics 19. Differences were considered statistically significant for values of *p* < 0.05.

## Figures and Tables

**Table 1:** The structures of the carbohydrate chains recognized especially by lectins.

Lectin	Full Name of the Lectin	Specificity Carbohydrate Chain
Jacalin	*Artocapusintegrifolia*	Galβ1-3GalNAcα-Ser/Thr(T), GalNAcα-Ser/Thr(Tn), GlcNAcβ1-3-GalNAcα-Ser/Thr(Core3), sialyl-T(ST). not bind to Core2, Core6, and sialyl-Tn (STn)
ECA	*Erythrinacristagalli*	Galβ-1,4GlcNAc (type II), Galβ1-3GlcNAc (type I)
HHL	*Hippeastrum Hybrid Lectin*	High-Mannose, Manα1-3Man, Manα1-6Man, Man5-GlcNAc2-Asn
WFA	*Wisteria Floribunda Lectin*	terminating in GalNAcα/β1-3/6Gal
GSL-II	*Griffonia (Bandeiraea) Simplicifolia Lectin II*	GlcNAc and agalactosylated tri/tetra antennary glycans
MAL-II	*Maackia Amurensis Lectin II*	Siaα2-3Galβ1-4Glc(NAc)/Glc, Siaα2-3Gal, Siaα2-3, Siaα2-3GalNAc
PHA-E	*Phaseolus vulgaris Agglutinin (E)*	Bisecting GlcNAc, biantennary complex-type N-glycan with outer Gal
PTL-I	*Psophocarpus Tetragonolobus Lectin I*	GalNAc, GalNAcα-1,3Gal, GalNAcα-1,3Galβ-1,3/4Glc
SJA	*Sophora Japonica Agglutinin*	Terminal in GalNAc and Gal, anti-A and anti-B human blood group
PNA	*Peanut Agglutinin*	Galβ1-3GalNAcα-Ser/Thr(T)
EEL	*Euonymus Europaeus Lectin*	Galα1-3(Fucα1-2)Gal(blood group B antigen)
AAL	*Aleuria Aurantia Lectin*	Fucα1-6 GlcNAc(core fucose), Fucα1-3(Galβ1-4)GlcNAc
LTL	*Lotus Tetragonolobus Lectin*	Fucα1-3Galβ1-4GlcNAc, Fucα1-anti-H blood group specificity
MPL	*Maclura Pomifera Lectin*	Galβ1-3GalNAc, GalNAc
LEL	*Lycopersicon Esculentum (Tomato) Lectin*	(GlcNAc)n, high mannose-type *N*-glycans
GSL-I	*Griffonia (Bandeiraea) Simplicifolia Lectin I*	αGalNAc, αGal, anti-A and B
DBA	*Dolichos Biflorus Agglutinin*	αGalNAc, Tn antigen, GalNAcα1-3((Fucα1-2))Gal (blood group A antigen)
LCA	*Lens Culinaris Agglutinin*	α-D-Man, Fucα-1,6GlcNAc, α-D-Glc
STL	*Solanum Tuberosum (Potato) Lectin*	trimers and tetramers of GlcNAc, core (GlcNAc) of N-glycan, oligosaccharide containing GlcNAc and MurNAc
PTL-II	*Psophocarpus Tetragonolobus Lectin II*	Gal, blood group H , T-antigen
DSA	*Daturastramonium*	(GlcNAc) 2-4, polyLacNAc and LacNAc (NA3, NA4)
VVA	*Vicia Villosa Lectin*	terminal GalNAc, GalNAcα-Ser/Thr(Tn), GalNAcα1-3Gal
MAL-I	*Maackia Amurensis Lectin I*	Galβ-1,4GlcNAc
GNA	*Galanthusnivalis*	High-Mannose, Manα1-3Man
NPA	*Narcissus Pseudonarcissus Lectin*	High-Mannose,Manα1-6Man
ACA	*Amaranthuscaudatus*	Galβ1-3GalNAcα-Ser/Thr (T antigen), sialyl-T(ST) tissue staining patterns are markedly different than those obtained with either PNA or Jacalin
BPL	*Bauhinia Purpurea Lectin*	Galβ1-3GalNAc, Terminal GalNAc
PHA-E+L	*Phaseolus vulgaris Agglutinin (E+L)*	Bisecting GlcNAc, bi-antennary N-glycans, tri- and tetra-antennary complex-type N-glycan
SNA	*Sambucus Nigra Lectin*	Sia2-6Gal/GalNAc
RCA120	*Ricinus Communis Agglutinin I*	β-Gal, Galβ-1,4GlcNAc (type II), Galβ1-3GlcNAc (type I)
BS-I	*Bandeiraeasimplicifolia*	α-Gal, α-GalNAc, Galα-1,3Gal, Galα-1,6Glc
PSA	*Pisum Sativum Agglutinin*	Fucα-1,6GlcNAc,α-D-Man, α-D-Glc
SBA	*Soybean Agglutinin*	α- or β-linked terminal GalNAc, (GalNAc)n, GalNAcα1-3Gal, blood-group A
WGA	*Triticum vulgaris*	Multivalent Sia and (GlcNAc)_n_
UEA-I	*Ulex Europaeus Agglutinin I*	Fucα1-2Galβ1-4Glc(NAc)
PWM	*Phytolaccaamericana*	(GlcNAc)_n_ and polyLacNAc
ConA	*Canavaliaensiformis*	High-Mannose, Manα1-6(Manα1-3)Man, αMannose, αGlc

## References

[1] Zhu Y., Lai B., Niu X., Wei J., Tan W., Wang X. (2014). Long-term prognostic value of major and minor ECG abnormalities in latent Keshan disease with suspect chronic Keshan disease. J. Epidemiol..

[2] He S., Tan W., Wang S., Wu C., Wang P., Wang B., Su X., Zhao J., Guo X., Xiang Y. (2014). Genome-wide study reveals an important role of spontaneous autoimmunity, cardiomyocyte differentiation defect and anti-angiogenic activities in gender-specific gene expression in Keshan disease. Chin. Med. J..

[3] Lei C., Niu X., Wei J., Zhu J., Zhu Y. (2009). Interaction of glutathione peroxidase-1 and selenium in endemic dilated cardiomyopathy. Clin. Chim. Acta.

[4] Li Q., Liu M., Hou J., Jiang C., Li S., Wang T. (2013). The prevalence of Keshan disease in China. Int. J. Cardiol..

[5] Li Y., Wen T., Zhu M., Li L., Wei J., Wu X., Guo M., Liu S., Zhao H., Xia S. (2013). Glycoproteomic analysis of tissues from patients with colon cancer using lectin microarrays and nanoLC-MS/MS. Mol. Biosyst..

[6] Jian Q., Yu H., Chen C., Li Z. (2009). Establishment of a Lectin Microarray Method for the Rapid Analysis of Glycoprotein and Its Application. Prog. Biochem. Biophys..

[7] Kautto L., Nguyen-Khuong T., Everest-Dass A., Leong A., Zhao Z., Willcox M.D., Packer N.H., Peterson R. (2016). Glycan involvement in the adhesion of Pseudomonas aeruginosa to tears. Exp. Eye Res..

[8] Sterba J., Vancova M., Sterbova J., Bell-Sakyi L., Grubhoffer L. (2014). The majority of sialylated glycoproteins in adult Ixodes ricinus ticks originate in the host, not the tick. Carbohydr. Res..

[9] Pilobello K.T., Slawek D.E., Mahal L.K. (2007). A ratiometric lectin microarray approach to analysis of the dynamic mammalian glycome. Proc. Natl. Acad. Sci. USA.

[10] Zhong Y., Qin Y., Yu H., Yu J., Wu H., Chen L., Zhang P., Wang X., Jia Z., Guo Y. (2015). Avian influenza virus infection risk in humans with chronic diseases. Sci. Rep..

[11] Liang Y., Ma T., Thakur A., Yu H., Gao L., Shi P., Li X., Ren H., Jia L., Zhang S. (2015). Differentially expressed glycosylated patterns of alpha-1-antitrypsin as serum biomarkers for the diagnosis of lung cancer. Glycobiology.

[12] Dassanayaka S., Jones S.P. (2014). O-GlcNAc and the cardiovascular system. Pharmacol. Ther..

[13] Mellor K.M., Brimble M.A., Delbridge L.M. (2015). Glucose as an agent of post-translational modification in diabetes—New cardiac epigenetic insights. Life Sci..

[14] Nagy T., Champattanachai V., Marchase R.B., Chatham J.C. (2006). Glucosamine inhibits angiotensin II-induced cytoplasmic Ca^2+^ elevation in neonatal cardiomyocytes via protein-associated *O*-linked *N*-acetylglucosamine. Am. J. Physiol. Cell Physiol..

[15] Marsh S.A., Powell P.C., Dell’italia L.J., Chatham J.C. (2013). Cardiac *O*-GlcNAcylation blunts autophagic signaling in the diabetic heart. Life Sci..

[16] Medford H.M., Porter K., Marsh S.A. (2013). Immediate effects of a single exercise bout on protein *O*-GlcNAcylation and chromatin regulation of cardiac hypertrophy. Am. J. Physiol. Heart Circ. Physiol..

[17] Chun W.J., Nah D.Y., Bae J.H., Chung J.W., Lee H., Moon I.S. (2015). Glucose-insulin-potassium solution protects ventricular myocytes of neonatal rat in an in vitro coverslip ischemia/reperfusion model. Korean Circ. J..

[18] Darley-Usmar V.M., Ball L.E., Chatham J.C. (2012). Protein *O*-linked beta-*N*-acetylglucosamine: A novel effector of cardiomyocyte metabolism and function. J. Mol. Cell. Cardiol..

[19] Maxwell J.T., Somasuntharam I., Gray W.D., Shen M., Singer J.M., Wang B., Saafir T., Crawford B.H., Jiang R., Murthy N. (2015). Bioactive nanoparticles improve calcium handling in failing cardiac myocytes. Nanomedicine.

[20] Muthusamy S., DeMartino A.M., Watson L.J., Brittian K.R., Zafir A., Dassanayaka S., Hong K.U., Jones S.P. (2014). MicroRNA-539 is up-regulated in failing heart, and suppresses *O*-GlcNAcase expression. J. Biol. Chem..

[21] Wang B., Tedder M.E., Perez C.E., Wang G., de Jongh Curry A.L., To F., Elder S.H., Williams L.N., Simionescu D.T., Liao J. (2012). Structural and biomechanical characterizations of porcine myocardial extracellular matrix. J. Mater. Sci. Mater. Med..

